# Porphyria Cutanea Tarda in a Patient with End-Stage Renal Disease: A Case of Successful Treatment with Deferoxamine and Ferric Carboxymaltose

**DOI:** 10.1155/2017/4591871

**Published:** 2017-01-22

**Authors:** Natacha Rodrigues, Fernando Caeiro, Alice Santana, Teresa Mendes, Leonor Lopes

**Affiliations:** ^1^Hemodialysis Unit Diaverum Cruz Vermelha Portuguesa, Lisbon, Portugal; ^2^Department of Dermatology, Centro Hospitalar Lisboa Norte, Lisbon, Portugal

## Abstract

*Porphyria cutanea tarda* (PCT) is a rare disease, with a strong association with hepatitis C virus. PCT is particularly problematic in end-stage renal disease patients as they have no renal excretion of porphyrins and these are poorly dialyzed. Also, conventional treatment of PCT is compromised in these patients as hydroxychloroquine is contraindicated, phlebotomies with the stipulated frequency are poorly tolerated in already anaemia-prone patients, and iron-chelating agents are less efficient in removing iron and contribute to worsening anaemia. The authors report a patient on haemodialysis, with hepatitis C infection, that is diagnosed with PCT. Despite the good clinical results with deferoxamine, she became dependent on blood transfusions because of her ferropenic state. Every time oxide iron was started, the patient developed clinical features of the disease, resolving after the suspension of the drug. A decision was made to start the patient on ferric carboxymaltose, which was well tolerated without disease symptoms and need of further blood transfusions. This case suggests that deferoxamine is efficient in treatment of porphyria cutanea tarda. Also, ferric carboxymaltose may be a valuable option for refractory anaemia in patients with this disease and end-stage renal disease, as it seems to provide iron without clinical relapse of the disease.

## 1. Introduction

Although dermatological manifestations in patients with end-stage renal disease (ESRD) can be very common, such as pruritus, xerosis, and pigmentation, other cutaneous disorders are quite rare [1]. Sporadic* porphyria cutanea tarda* (PCT) is such a disease, occurring in less than 5% of dialysis patients [2]. Despite the low prevalence, there is a strong association between the sporadic form of PCT and hepatitis C virus (HCV) infection that has been demonstrated in multiple studies [3].

Although rare, PCT has particular issues in ESRD patients. Not only do these patients have no renal excretion of porphyrins and these are poorly dialyzed, but also although the treatment of PCT in the general population is well established, in ESRD patients hydroxychloroquine is contraindicated, frequent phlebotomies tend to be poorly tolerated by these already anaemia-prone patients, and iron-chelating agents are less efficient in removing iron and contribute to worsening anaemia.

## 2. Case Report

We report the case of a 46-year-old melanodermic woman with end-stage renal disease of unknown aetiology. She started haemodialysis (HD) in 1996 and received a cadaveric kidney transplant in 1997. After transplantation, she had cytomegalovirus infection, colon adenocarcinoma, and chronic renal allograft dysfunction. She restarted haemodialysis in 2003 due to graft dysfunction.

She also had HCV infection (genotype 1a) and she was put on pegylated interferon-alpha-2a 180 mcg/week from July 2010 to July 2011; however, a negative viral load was not attained with this treatment regimen.

**Figure 1:**
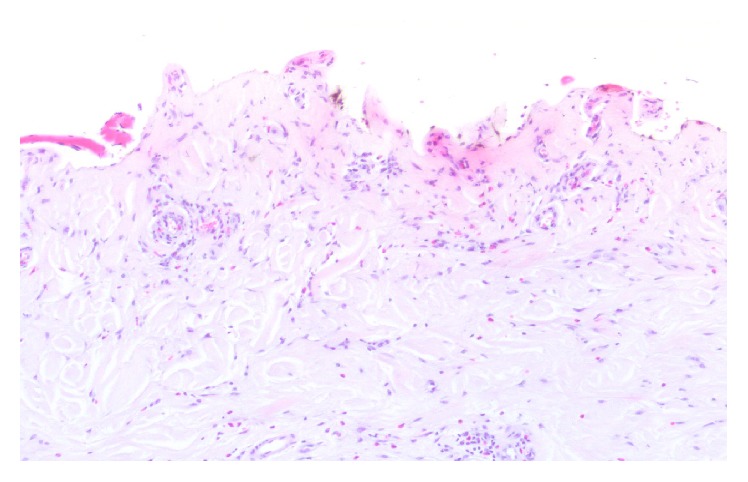
Cutaneous biopsy tissue. Skin biopsy specimens showing epidermal detachment with minimal dermal inflammatory infiltrate, festooning of dermal papillae with thickening of basement membrane, and deposits of eosinophilic hyaline material (PAS positive) on the wall of superficial vascular plexus (H&E 100x).

In March of 2014, she presented with bullous dermatosis evolving both upper limbs, more exuberant on the dorsal aspect of the hands and forearms, and oral enanthema more prominent in the lower lip, interfering with meals. Despite her skin and mucosal lesions, physical examination was otherwise unremarkable. She did not have any family history of porphyria, photosensitivity, or prior episodes of this dermatosis. She was not on oestrogen replacement therapy or had a history of alcoholism. At this time, she was on epoetin 160 U/kg/week IV, alfacalcidol 1 mcg/HD session, Generis saccharated iron oxide 50 mg/week, and carvedilol 12,5 mg/bid. The lesions were biopsied: epidermal detachment with minimal dermal inflammatory infiltrate, festooning of dermal papillae with thickening of basement membrane, and deposits of eosinophilic hyaline material on the wall of superficial vascular plexus (Figure 1).

The laboratory investigation is presented in Table 1 and showed an appropriate haemoglobin, normal white blood cell count, platelet count, C-reactive protein level, transferrin saturation, ferritin, alanine aminotransferase, aspartate aminotransferase, and slightly increased gamma-glutamate transpeptidase. Serologic tests were negative for HIV and hepatitis B and were positive for hepatitis C. The patient had elevated levels of total plasma porphyrin, uroporphyrin, and heptacarboxylporphyrin and hexacarboxylporphyrin; she had normal levels of pentacarboxylporphyrin, coproporphyrin I, and coproporphyrin II. Faecal porphyrin levels were normal. Urinary porphyrins were not measured because she was anuric.

With these findings, she was diagnosed with porphyria cutanea tarda. Phlebotomy was not an option considering the anaemia secondary to chronic renal disease in a patient already under a significant dose of epoetin. Generis saccharated iron oxide was suspended and she was started on deferoxamine 4 mg/kg/week (intravenous infusion in the end of dialysis session) for 6 weeks. Skin lesions disappeared and laboratorial revaluation showed haemoglobin of 98 g/L, transferrin saturation of 16%, and ferritin of 140 *μ*g/L.

During the subsequent months, a progressive drop in haemoglobin levels was registered, despite augmentation of epoetin dose to 600 U/kg/week. She became symptomatic with fatigue; her haemoglobin was 77 g/L, transferrin saturation was 6%, and ferritin was 16 *μ*g/L. A red blood cell transfusion was administered and despite the transitory response repeated transfusions were needed. After one year, it was decided to restart Generis saccharated oxide iron 50 mg/week; however, skin lesions returned with the previous pattern. Generis saccharated oxide iron was suspended, resulting in skin lesion remission and as her anaemia became symptomatic, the patient maintained the need for regular red blood cell transfusions. This prompted the medical team to search for alternative iron therapies in order to avoid the need for repeated red blood cell transfusions and surpass the recurrent lesions which appeared to be linked to the use of saccharated oxide iron. Ferric carboxymaltose was administered in a dose of 500 mg and the patient tolerated this treatment with no skin lesions, ferritin elevation to 49 *μ*g/L, and increased response to epoetin. No more red blood cell transfusions were required. The administration of ferric carboxymaltose was repeated after two months with good results. The patient is now medicated with 120 U/kg/week epoetin, has haemoglobin of 114 g/L, and has started HCV treatment with direct acting antivirals.

## 3. Discussion

Deficiency or altered activity of enzymes responsible for heme biosynthetic pathway leads to a group of diseases nominated porphyrias. Clinical manifestations are predominantly visceral or cutaneous, depending on the affected enzyme and what step of the pathway is compromised, resulting in both the accumulation of some certain porphyrins and the deficit of others. The most common of these rare disorders is porphyria cutanea tarda (PCT) caused by an activity deficit of the fifth enzyme in the heme synthetic pathway uroporphyrinogen decarboxylase (UROD) [4]. Genetic factors can contribute to increased susceptibility, namely, heterozygosity for UROD mutation, reducing activity to 50% of normal activity, or hemochromatosis mutations, increasing iron absorption. Acquired factors are well known such as alcohol, smoking, oestrogen, human immunodeficiency virus, hepatitis C virus, and secondary causes of iron overload. The precise mechanism by which HCV infection might cause or act as a trigger for PCT in predisposed subjects is not known but is thought to be related to alterations in iron metabolism [5]. Patients on haemodialysis may be prone to PCT by their occasional exposure to iron overload states through intravenous iron, blood transfusions, and the accumulation of porphyrins not totally removed by the dialytic technique [6].

The diagnosis of PCT implies dermatologic manifestations such as itching and painful skin lesions, chronic photosensitivity with blisters, bullae, scarring, increased skin fragility, and hyper-hypopigmentation, affecting sun-exposed areas, hirsutism, and alopecia. It also implies increased plasma or urine porphyrins formed before the UROD step such as uroporphyrin, heptacarboxylporphyrin, hexacarboxylporphyrin, and pentacarboxylporphyrin (urine analysis not validated for patients with end-stage renal disease (ESRD)) with concomitant normal levels of coproporphyrins [7].

The first-line treatment for PCT is phlebotomy with removal of 450 mL every two weeks and low-dose hydroxychloroquine along with identifying and minimising predisponent factors present in each patient and skin protection from sunlight until porphyrin levels have normalised [8]. Unfortunately, patients with ESRD present specificities that make treatment a true challenge. Not only is hydroxychloroquine contraindicated in ESRD, but also phlebotomies are normally not tolerated in already anaemia-prone patients as well as in patients with poor venous access. Iron-chelating agents are less efficient in removing iron than phlebotomy [9] but have been proposed as an option in ESRD with anaemia.

The administration of iron-chelating agents, namely, deferoxamine, for the treatment of PCT allowed remission in our patient, although our patient did not have a very high ferritin concentration, corroborating the hypothesis of this drug being a good option for patients with ESRD and anaemia.

The consequent ferropenic status after the treatment with deferoxamine contributed to the refractory anaemia developed by the patient afterwards and she became symptomatic. On one hand, red blood cell transfusions contain not only young but also old erythrocytes, meaning that a considerable portion of them will be caught by the spleen in a short time after transfusion and the erythrocytes' catabolism will result in production of porphyrins, which is not obviously desirable in a patient suffering of PCT. On the other hand, in a patient with ESRD, ferropenic status, and symptomatic anaemia under already maximized erythropoiesis-stimulating agent therapy and with known association of trigger of PCT with previous iron administration, we considered red blood cell transfusion as an appropriate therapy on that moment.

In an attempt to end with the dependency of red blood cell transfusions, saccharated oxide iron was reintroduced and resulted in relapse of PCT. The suspension of saccharated oxide iron was sufficient to achieve remission; however, the ferropenic state contributed considerably to dependence on red blood cell transfusions, leading the authors to try a different approach and to administer ferric carboxymaltose with success. Although the rationale for ferric carboxymaltose not precipitating PCT relapse is not fully understood, the answer is certainly related to the fact that different iron carbohydrate complexes have different pharmacokinetic/pharmacodynamic characteristics and specific reactivities. The success of this case may be related to the fact that, compared to saccharated ferric oxide, ferric carboxymaltose is a more stable, large sized complex formulated as a colloidal solution with physiological pH and therefore releases less amounts of iron at a time over a long period of time [10], which might result in less saturation of the heme pathway with less accumulation of porphyrins and is therefore not enough to trigger PCT manifestations. Studies are necessary to validate this hypothesis.

## 4. Conclusion

Deferoxamine is an efficient treatment for porphyria cutanea tarda in end-stage renal disease patients. Furthermore, ferric carboxymaltose may be a valuable option for refractory anaemia in these patients, as it seems to provide iron without clinical relapse of the disease conversely to other iron formulations. However, studies are necessary to validate this hypothesis.

## Figures and Tables

**Table 1:** Laboratory results.

	Result	Reference
Haemoglobin	104 g/L	100–120 g/L
WBC count	6.06 × 10^9^/L	3.8–10.8 × 10^9^/L
Platelet count	138 × 10^9^/L	150–450 × 10^9^/L
C-reactive protein	0.04 mg/L	<0.8 mg/L
Transferrin saturation	43%	>30%
Ferritin	267 *µ*g/L	200–500 *µ*g/L
ALT	53 U/L	<20 U/L
AST	34 U/L	<42 U/L
G-GT	133 U/L	8–65 U/L
Total plasma porphyrin	1052.3 ug/L	<32.5 ug/L
Plasma uroporphyrin	983.3 ug/L	< 11.8 ug/L
Plasma heptacarboxylporphyrin	24.3 ug/L	<3.8 ug/L
Plasma hexacarboxylporphyrin	13.4 ug/L	<1.3 ug/L
Plasma pentacarboxylporphyrin	27.3 ug/L	<27.3 ug/L
Plasma coproporphyrin I	2.5 ug/L	<6.4 ug/L
Plasma coproporphyrin II	1.5 ug/L	<8 ug/L
Faecal porphyrins	All normal	
Urinary porphyrins	Anuric	

ALT, alanine aminotransferase; AST, aspartate aminotransferase; G-GT, gamma-glutamate transpeptidase. The reference values for haemoglobin, ferritin, and transferrin saturation are in concordance with KDIGO considering ESRD patients.
